# Enhanced In Vitro Magnetic Cell Targeting of Doxorubicin-Loaded Magnetic Liposomes for Localized Cancer Therapy

**DOI:** 10.3390/nano10112104

**Published:** 2020-10-23

**Authors:** Eugenio Redolfi Riva, Edoardo Sinibaldi, Agostina Francesca Grillone, Serena Del Turco, Alessio Mondini, Tianshu Li, Shinji Takeoka, Virgilio Mattoli

**Affiliations:** 1 The BioRobotics Institute and Department of Excellence in Robotics and AI, Scuola Superiore Sant’Anna, 56124 Pisa, Italy; 2 Center for Micro-BioRobotics, Istituto Italiano di Tecnologia, Viale Rinaldo Piaggio 34 Pontedera, 56025 Pisa, Italy; agostina.grillone@iit.it (A.F.G.); alessio.mondini@iit.it (A.M.); 3 Institute of Clinical Physiology, CNR San Cataldo Research Area, Via Giuseppe Moruzzi 1, 56124 Pisa, Italy; serena@ifc.cnr.it; 4 Institute for Advanced Research of Biosystem Dynamics, Research Institute for Science and Engineering, Waseda University, Tokyo 169-8050, Japan; tianshuli@aoni.waseda.jp (T.L.); takeoka@waseda.jp (S.T.); 5 School of Advanced Science and Engineering, Waseda University, Tokyo 169-8050, Japan

**Keywords:** magnetic drug targeting, magnetic liposomes, nanomedicine

## Abstract

The lack of efficient targeting strategies poses significant limitations on the effectiveness of chemotherapeutic treatments. This issue also affects drug-loaded nanocarriers, reducing nanoparticles cancer cell uptake. We report on the fabrication and in vitro characterization of doxorubicin-loaded magnetic liposomes for localized treatment of liver malignancies. Colloidal stability, superparamagnetic behavior and efficient drug loading of our formulation were demonstrated. The application of an external magnetic field guaranteed enhanced nanocarriers cell uptake under cell medium flow in correspondence of a specific area, as we reported through in vitro investigation. A numerical model was used to validate experimental data of magnetic targeting, proving the possibility of accurately describing the targeting strategy and predict liposomes accumulation under different environmental conditions. Finally, in vitro studies on HepG2 cancer cells confirmed the cytotoxicity of drug-loaded magnetic liposomes, with cell viability reduction of about 50% and 80% after 24 h and 72 h of incubation, respectively. Conversely, plain nanocarriers showed no anti-proliferative effects, confirming the formulation safety. Overall, these results demonstrated significant targeting efficiency and anticancer activity of our nanocarriers and superparamagnetic nanoparticles entrapment could envision the theranostic potential of the formulation. The proposed magnetic targeting study could represent a valid tool for pre-clinical investigation regarding the effectiveness of magnetic drug targeting.

## 1. Introduction

Hepatocellular carcinoma (HCC) is the most common liver tumor occurring in human beings. It is the sixth most frequent cancer worldwide and the second leading cause of cancer-related death: in 2012 over 700,000 people worldwide died of liver cancer [1,2]. HCC mostly affects men and its incidence increases rapidly with age [3]. Therapeutic interventions depend on the volume of the primary cancerous mass and particularly on the quantity of tumor metastasis in other organs. When surgical resection is not feasible, chemotherapy is the gold standard, in association with radiotherapy [4]. Lots of chemotherapeutic drugs have been approved against HCC: tyrosin kinase inhibitors, such as sorafenib and sunitinib, DNA-targeted molecules as cisplatin and selective estrogen-receptor modulator as tamoxifen [5,6,7,8]. Moreover, Doxorubicin (DOX), which is a topoisomerase II inhibitor, has evidenced its efficacy against HCC correlated with relatively moderate side effects with respect to other chemotherapeutic drugs [9,10]. Although the efficacy of some chemotherapeutic drugs against HCC growth have been proved, severe side effects have been reported, such as proteinuria, skin related toxicities, an increased risk for thromboembolism, and bleeding events [11]. This occurrence not only has a crucial impact on patient’s quality of life, but, in some cases, impede drug treatment applicability. In this regard, it is clear how solutions that would improve drug safety and efficacy are of primary clinical interest.

In the last decades, nanotechnology has paved the way to multiple solutions for improving drug specificity and therapeutic safety, providing various nanocarriers to enhance drug biodistribution and minimize drug dispersion and side effects [12,13,14,15,16]. Lipid nanocarriers have been studied for several years in the field of nanomedicine, owing to their excellent biocompatibility and high drug payload [17,18,19]. In the literature, several examples of this class of nanocarriers have been studied for drug encapsulation and gene therapy purposes. One of the most interesting subsets of lipid nanocarriers is unilamellar lipid vesicles, usually called liposomes [20,21].

However, a common bottleneck is still present in the field of lipidic nanoparticles drug delivery and, more generally, in nanomedicine: the difficulty in obtaining an efficient targeting of the interested site [22,23,24]. Physiological barriers could retain nanoparticles, thus decreasing the available therapeutic amount [25]. In this scenario, it is clear how nanoparticles targeting capability in systemic administrated nanovectors is a crucial issue that needs to be deeply investigated, especially for cancer therapy [26].

In this vision, the encapsulation of superparamagnetic iron oxide nanoparticles inside liposomes could be an advantageous strategy to alter liposome biodistribution upon the application of an external magnetic field, thus enhancing their cellular uptake towards a selected area, avoiding drug dispersion. Some examples are present in literature regarding magnetic nanoparticles and liposomes as theranostic platforms for controlled drug delivery, for MRI contrast enhancement and for hyperthermia [17,18,19,20,21,22,23,24,25,26,27,28,29,30,31]. Magnetic nanoparticles incorporation inside liposomes could be exploited for magnetic assisted drug delivery (MDD), in order to increase particles accumulation nearby cancer lesion [32,33]. Moreover, recent works on MDD have focused on combining multiple approaches to drug delivery in one nanocarriers, such as ultrasound-triggered drug release, photo-thermal treatment, and photodynamic therapy upon enhanced particles cell uptake [34,35,36,37,38,39,40,41]. However, few studies regarding MDD focused on in vitro analysis of magnetic targeting efficacy in dynamic environment, resulting in current difficulties for improving the clinical translation of nanocarriers [42]. The study of Martina et al., [43] on the effect of magnetic targeting on cell internalization of magnetic liposomes is worth mentioning. They studied intracellular trafficking of their magnetic liposomes formulation upon application of an external magnetic field. However, these experiments have been conducted in static conditions without assessing liposomes cell uptake under different dynamic environmental conditions. Another relevant study is the recent work of Szuplewska and colleagues regarding the cytotoxicity assessment of DOX-loaded magnetic liposomes towards human breast cancer cells [44]. They presented an in vitro characterization of enhanced DOX release and cytotoxicity after exposition to alternating magnetic field. However, liposomes magnetic cell targeting capability and internalization under cell medium flow has not been investigated.

Here, we present a stable DOX-loaded Magnetic/Lipidic Nanocarrier as a possible theranostic tool against HCC. In this study, we decided to focus on in vitro assessment of targeting efficacy of our formulation under flow conditions and an external magnetic field, as insufficient nanoparticles cancer cell targeting has been reported to be one of the main issues that restricts clinical translation of cancer nanomedicine [45,46,47]. This represents the main novelty of the work and we believe that this approach could represent a useful tool for improving nanocarriers cell targeting in order to foster their use for patient’s healthcare. The incorporation of magnetic nanoparticles aims to enhance liposomes uptake in correspondence of the tumor site upon the application of an external magnetic field. As this regard, an in vitro investigation on the enhanced nanoparticles uptake in the presence of an external magnetic field at different flow conditions was presented and compared with a numerical model. This characterization was performed with two cell lines, in order to evaluate selective liposomes tumor cells uptake (using HepG2 cells) and validate experimental data on magnetic targeting (using endothelial cell line). Furthermore, magnetic liposomes cytotoxicity on a human hepatocellular carcinoma cell line (HepG2) was studied, as well as DOX release kinetic under different environmental conditions and liposomes localization inside cell cytoplasm upon internalization.

This analysis can be used in order to further assess the proposed MDD targeting strategy and properly tune formulation parameters prior to further in vivo characterization, in view of potential clinical translation.

## 2. Materials and Methods

### 2.1. Materials

Dipalmitoylphosphatidylcholine (DPPC), plastic dialysis tubes (Pur-A-Lyzer, membrane cutoff = 3.5 kDa), Hepatocellular carcinoma cell line (HepG2), and cell culture reagents were supplied by Sigma–Aldrich (St. Louis, MO, USA). Cholesterol (Chol) was purchased by Avanti Polar Lipids (Alabaster, AL, USA). 1,5-O-dihexadecyl-*N*-succinyl-L-glutamate1,5-O-dihexadecyl-*N*-succinyll-glutamate (DHSG) and 1,2-distearoyl-*sn*-glycero-3-phosphoethanolamine-N-(polyethylene glycol)-5000 (DSPE-PEG_5000_) were obtained by Nano CS (Boston, MA, USA). Dextran-coated magnetic nanoparticles (MNPs) water dispersion (nanomag^®^-D-spio, product code 79-00-102) was supplied by Micromod Partikeltechnologie GmbH (Rostock, Germany). Sephadex G-25 columns was purchased by GE Healthcare Life Sciences (Milano, Italy). Doxorubicin hydrochloride (DOX), Topoisomerase II inhibitor was supplied by Abcam (Cambridge, UK). Cholesterol E-test kits were purchased from WAKO Pure Chemical Industries, Ltd. (Osaka, Japan). Plastic µ-slides (µ-Slide VI0.4) were supplied by Ibidi (Gräfelfing, Germany)). Fluorescent lipophylic dye (DiO) was purchased by Life Technologies (Carlsbad, CA, USA). Phalloidin and DAPI were purchased by Millipore (Burlington, MA, USA). WST-1 assay kit (2-(4-iodophenyl)-3-(4-nitophenyl)-5-(2,4-disulfophenyl)-2H-tetrazoilium monosodium salt) was supplied by BioVision (Zürich, Switzerland).

### 2.2. Methods

#### 2.2.1. Cell Culture

HepG2 cells (Sigma–Aldrich, St. Louis, MO, USA) were cultured in low glucose Dulbecco’s Modified Eagle Medium (DMEM) containing 10% fetal bovine serum (FBS), 1% non-essential amino acid solution (NEAAS), and 1% penicillin-streptomycin, at 37 °C with 5% CO_2_ atmosphere. Human umbilical vein endothelial cells (HUVECs) were harvested and isolated, as previously described [48]. Human cells were obtained from discarded umbilical cords and treated anonymously. As such, approval from the University Ethics Review Board was not necessary. The cells have been cultured in Medium 199 containing 10% of fetal bovine serum (FBS), 2 mmol/L glutamine, 20 ng/mL of ECGF, and 1% of penicillin-streptomycin, at 37 °C with 5% CO_2_ atmosphere. All of the experiments were performed with low-serum cell culture medium.

#### 2.2.2. Magnetic Liposomes Fabrication

Magnetic liposomes (MagLipo) were fabricated while using a thin-film evaporation method. In particular, 10 mg lipid powder composed with DPPC, cholesterol(Chol), DHSG, and DSPE-PEG_5000_ with a molar ratio of DPPC/Chol/DHSG/DSPE-PEG_5000_ = 5/4.85/1.04/0.03 was dissolved in chloroform using a round bottom flask and put under vacuum overnight for complete solvent dissolution, in order to obtain a thin lipid film onto the bottom of the flask.

Subsequently, the thin lipid film was hydrated under stirring for 12 h with 10 mL of a 2 mg/mL dextran-coated magnetic nanoparticles (MNPs) aqueous dispersion with a 20 nm hydrodynamic diameter, in order to obtain multilamellar lipid vesicles (MLV). After this procedure the MLV aqueous dispersion was extruded with a manual system (Avanti Polar Lipid, Alabaster, AL, USA) that was composed of two syringes that force the dispersion to pass through a polycarbonate membrane filter with a pore size of 0.2 µm. Eleven passages through the membrane were used in order to obtain liposomes that contain magnetic nanoparticles. Finally, MagLipo dispersion was purified while using Sephadex G-25 pre-packed columns with deionized water as eluent, in order to remove free magnetic nanoparticles from the dispersion.

**Figure 1:**
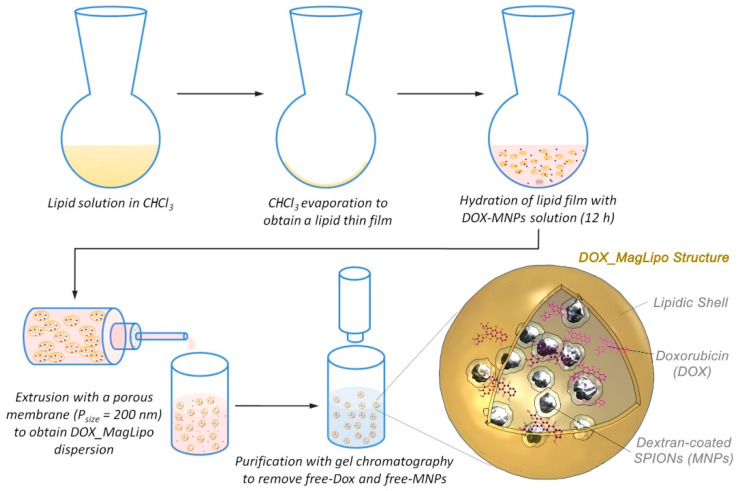
DOX_MagLipo fabrication and purification process and schematic structure. For MagLipo fabrication a 2 mg/mL MNPs solution without DOX was used.

DOX-loaded magnetic liposomes (DOX_MagLipo) were obtained following the same procedure as for MagLipo, hydrating the thin lipid film with 5 mL of a 2 mg/mL MNPs dispersion containing a DOX concentration of 1 mg/mL. DOX loading inside MagLipo was performed during the hydration process of the thin lipid film instead of using the common pH-gradient method [49]. This choice was made because of the tendency of dextran-coated MNPs to aggregate and precipitate under highly acidic environments required to perform the pH-gradient method that would have compromised both MNPs encapsulation inside lipid vesicles and phospholipidic bilayer formation. Purification was performed while using Sephadex G-25 pre-packed columns with deionized water as eluent, in order to remove the free DOX and the free magnetic nanoparticles from the solution (Figure 1). All of the samples have been stored in the refrigerator at 4 °C to prevent DOX leaching from liposomes.

#### 2.2.3. Characterization of Magnetic Liposomes

Morphology of DOX_MagLipo and MagLipo was analyzed using Atomic Force Microscopy (AFM) (Innova SPM, Bruker, Billerica, MA, USA), by drying a water dispersion of diluted liposomes dispersion onto a silicon substrate. AFM scans were performed in a tapping mode, by using NT-MDT NSG01 antimony-doped n-type silicon probes with a resonance frequency of 87-230 kHz and a force constant of 1.45–15.1 N.m^−1^. All data were elaborated and analyzed with a Gwyddion SPM analysis tool (http://gwyddion.net). Moreover, scanning transmission electron microscopy (S/TEM) images of MagLipo were performed in order to investigate the presence of encapsulated MNPs in the liposome, using a dual-beam system (FEI Helios 600, Hillsboro, OR, USA).

The size and surface charge of DOX_MagLipo and MagLipo were evaluated by means of dynamic light scattering using a Zetasizer Nano S90 (Malvern, Worcestershire, United Kingdom). Lipid concentration of liposomes was measured using a cholesterol E-test kit according to the manufacturer’s instructions. The MNPs concentration was measured by dissolving lipid bilayer with a 5% solution of TritonX-100, by monitoring the absorbance at 500 nm and correlating it to a standard calibration curve prepared with several concentrations of the same MNPs encapsulated inside liposomes. DOX loading into magnetic liposomes was measured by fluorescence spectroscopy using a microplate reader (Victor3, Perkin Elmer), setting the excitation wavelength at 480 nm and by acquiring fluorescence signal from 560 to 590 nm. DOX fluorescence intensity was then correlated to the drug concentration using a standard calibration curve.

Magnetic characterization of MagLipo was carried out using a superconducting quantum interference device (SQUID) from Quantum Design (MPMS-7, Quantum Design, San Diego, CA, USA). In order not to waste DOX, only MagLipo and MNPs were tested, since the presence of the drug inside liposome does not alter the magnetization of samples. After lyophilization of the liposomes, the dried powder was encapsulated into a gelatin capsule and placed in a straw for the SQUID analysis. Magnetization curves were measured from −30 to 30 kOe at 5 K and 310 K for both MagLipo and MNPs.

#### 2.2.4. Magnetic Targeting Experiments

**Figure 2:**
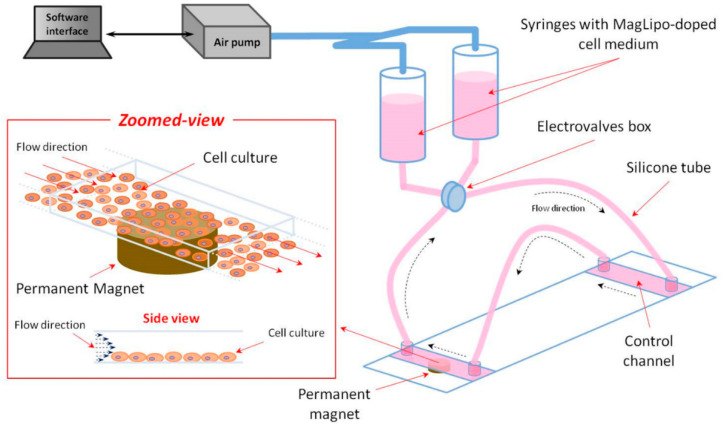
Schematization of the set-up for magnetic targeting experiments.

Magnetic targeting experiments were performed with MagLipo while using a dynamic perfusion set-up in order to investigate the enhancement of cell internalization of particles upon exposure to an external magnetic field. This system was composed by two syringes that were linked to a fluidic unit with programmable electro valves and plastic µ-slides (µ-Slide VI^0.4^, Ibidi) for cell culturing (Figure 2).

This system was reported in our previous study [50]. Briefly, a home-made air pump injects an air flow inside two syringes that were connected to two cell seeded microfluidic channels, pushing a MagLipo-doped cell culture medium inside the microfluidic channels with a selectable flow rate. Air pressure control was guaranteed by an ad hoc close loop system, which was connected with a custom software interface. Magnetic targeting experiments was performed by seeding microfluidic channels with two different cell lines: HepG2 cells to investigate MagLipo enhanced magnetic cancer cell uptake under flow conditions, and HUVECs in order to analytically assess targeting efficacy both under constant and pulsatile flow conditions. Endothelial cells are a useful model for studying the targeting ability of MagLipo in a linear blood vessel and they are the first barrier that nanocarriers meet after an intravenous route before reach the tumor mass.

In order to evaluate the amount of MagLipo internalized inside cells, MagLipo were stained before magnetic targeting experiments with DiO lipophilic dye by incubation at 37 °C for 1 h (1 mL MagLipo + 5 µL DiO) and subsequently dialyzed overnight (cutoff Mw 35 kDa) versus DI water to remove free dye from MagLipo dispersion. In order to calculate the quantity of MagLipo for experiments, an aliquot of known volume of liposome dispersion was freeze-dried overnight and weighted. Upon staining, MagLipo dispersion were mixed with cell culture medium and loaded in the perfusion set-up for magnetic targeting experiments. At the end of these experiments, cells were fixed and stained and then images were taken via confocal microscopy (C2s, Nikon). The fluorescent signal from MagLipo is proportional to the quantity of nanoparticles internalized inside cells.

##### Selective Cancer Cells Internalization in Flow Conditions: HepG2 Experiments

The HepG2 cells were seeded at the bottom of two rectangular-shaped microfluidic channels of the µ-Slide (7 × 10^4^ cells per each channel) and then incubated for three days at the same conditions to reach confluence. All of the seeded channels were incubated with gelatin (4%) for 20 min., at 37 °C and washed three times with PBS to improve cell adhesion. Under one of the channels a permanent neodymium magnet (*Br* = 1.32 T) of 6 mm diameter and 6 mm thickness was placed at a distance of 1.2 mm from the bottom of the microfluidic channel. All of the experiments with HepG2 cancer cells were performed for 3 h using a DiO-stained MagLipo concentration of 100 μg/mL, using a constant flow rate of about 2 mL/min. corresponding to a wall shear stress of about 4 dyn/cm^2^. These values were chosen in order to prevent HepG2 detachment from the microchannel during the exposure of cell medium flow. At the end of the experiments, the equipment was stopped and cells were rinsed with PBS, fixed with paraformaldehyde (4% in PBS), treated with 0.1% Triton X-100 to allow membrane permeation, and blocked for 1 h with a goat serum solution (10% in PBS). After these procedures, the cell cultures were incubated with a staining solution containing phalloidin (1:100) and DAPI (1:1000) to label cytoskeletal f-actin and nuclei, respectively. After thorough PBS rinsing, the images were acquired via confocal microscopy. Software image analysis using Fiji ImageJ software (https://imagej.net/Fiji) was performed in order to calculate average MagLipo quantity inside HepG2 cells and the data were reported as the percentage of area occupied by liposomes over the average scan area occupied by cells cytoplasm.

##### Numerical Description of Magnetic Targeting: HUVECs Experiments

Magnetic targeting experiments were also performed in order to compare MagLipo enhanced cell internalization with a numerical model of accumulation process in the presence of a defined flow. HUVEC cells were selected for these experiments, since they represent the first barrier that MagLipo meet after the intravenous route and also because of their stronger adhesion to the bottom of the microchannel respect to HepG2 cells. All of the seeded channels were incubated with gelatin (4%) for 20 min. at 37 °C and washed three times with PBS in order to improve cell adhesion. HUVECs (7 × 10^4^ cells per each channel) were cultured in Medium199 in order to reach confluence before experiments. HUVEC experiments were performed for 4 h using a DiO-stained MagLipo concentration of 100 μg/mL, using a constant flow rate of 3.11 mL/min. with a wall shear stress value of 5.7 dyn/cm^2^. This value was chosen according to literature [51,52]. A permanent neodymium magnet of 6 mm diameter and 6 mm thickness (with magnetization *B_r_* = 1.32 T) was placed at the bottom of one of the seeded channels to magnetically target MagLipo near the area of action of the magnetic field, while the other channel was used as the negative control. Thereafter, at the end of the experiment cells were washed and stained using the same procedure explained for HepG2 experiments. For HUVECs experiments the whole cell-seeded microchannel area was acquired via confocal microscopy by automatically moving microscope sample stage around the channel. In this way, the microchannel area was acquired as a 64 × 16 matrix of fluorescence images. Fluorescence signal reconstruction of the channel area was performed while using an NIS-element viewer (https://www.microscope.healthcare.nikon.com) and the stitching tool of Fiji ImageJ software (https://imagej.net/Fiji).

We also studied the aforementioned magnetic targeting problem using the numerical model that was introduced in literature [53]. The considered flow channel had a rectangular cross-section (3.8 mm width, 0.4 mm height) and the flow was uniform (thus considered to be fully developed) over a 17 mm span. The velocity field in the cross-section was analytically derived based on potential-flow methods (thus permitting computing the aforementioned wall shear stress starting from the input flow rate, assuming 10^−3^ Pa s viscosity, as for water). MagLipo were assumed to be dragged by the flow (one-way coupling, based on a classical Stokes model) and subjected to the magnetic attraction that was caused by the external magnet, as point dipoles (also accounting for magnetization saturation [54]). Given the liposome volume (based on the aforementioned average radius), the volume fraction that was occupied by MNPs was around 2.9 × 10^−3^ based on the experimentally measured iron mass fraction (6.3%, by ICP-MS on lyophilized sample). Moreover, liposome saturation magnetization was consistently derived from that one per unit iron mass of the MNPs (69 emu/g_iron_, from datasheet); it resulted in being around 2.6 × 10^5^ A/m. Furthermore, the permanent magnet was modeled by the classical currents method (with 10^3^ current strips: a number high enough to obtain discretization-independent results). The trajectory of 1.44 × 10^6^ liposomes in silico seeded upstream the magnet was numerically integrated in time (by using the Matlab numerical environment) up to impinging on either the channel floor (capture condition) or the outflow cross-section (escape condition). The captured carriers were then binned over a 64 × 16-cell grid in order to render the capture density through a contour plot (in arbitrary units) corresponding to the aforementioned experimental fluorescence recordings.

Moreover, magnetic targeting experiments with HUVECs were also performed under pulsatile flow conditions to assess MagLipo targeting capabilities, even under more critical environmental conditions. The experimental procedure and results of this characterization are shown in the Supporting Information section (Figure S2).

#### 2.2.5. Nanocarriers Internalization and Cytotoxicity Experiments on Hepatocellular Carcinoma Cell Line

Confocal microscopy was used in order to investigate MagLipo internalization by HepG2. The cells were seeded at a density of 12 × 10^3^/cm^2^ on ibidi µ-Dish (35 mm, Ibidi) and they were incubated at 37 °C and 5% CO_2_ in order to grow in confluence. MagLipo were stained in green with a lipophilic fluorescent dye (DiO; Life Technologies), for labeling hydrophobic structures, and consequently added to the cell culture medium at a concentration of 100 µg/mL. After an incubation of 24 h, the cells were rinsed with PBS and treated for 45 min. with LysoTracker (Invitrogen), a fluorescent probe for labeling acidic organelles (lysosomes) in live cells. Finally, the cells were rinsed with PBS, incubated for 10 min. with Hoechst 33342 (5 µg/mL, H1399 Invitrogen) for nucleus staining, and observed with a confocal microscope (C2s, Nikon).

The biocompatibility of MagLipo and cytotoxic effects of free DOX and of DOX_MagLipo against HepG2 cancer cells were evaluated using a WST-1 assay. For these experiments cells were seeded at a density of 3 × 10^4^ cells/well in 24-well plates and then incubated for 72 h at 37 °C and 5% CO_2_ in order to grow in confluence. Hence, the cell medium was replaced with fresh medium containing increasing concentrations of MagLipo (0, 10, 20, 50, 100, and 200 µg/mL), DOX_MagLipo (0, 10, 20, 50, 100, and 200 µg/mL), or free DOX at the same equivalent drug concentrations to those present inside DOX_MagLipo (0, 0.128, 0.257, 0.647, 1.28, 2.58 µM). Cell viability was assessed after 24 h and 72 h again using the WST-1 assay: cell culture medium was replaced with 300 µL of medium +30 µL of the pre-mix solution, and then incubated for 1 h. Finally, the absorbance has been read at 450 nm with a microplate reader (Victor3, Perkin Elmer, Waltham, MA, USA).

## 3. Results and Discussion

### 3.1. Magnetic Liposomes Characterization

**Figure 3:**
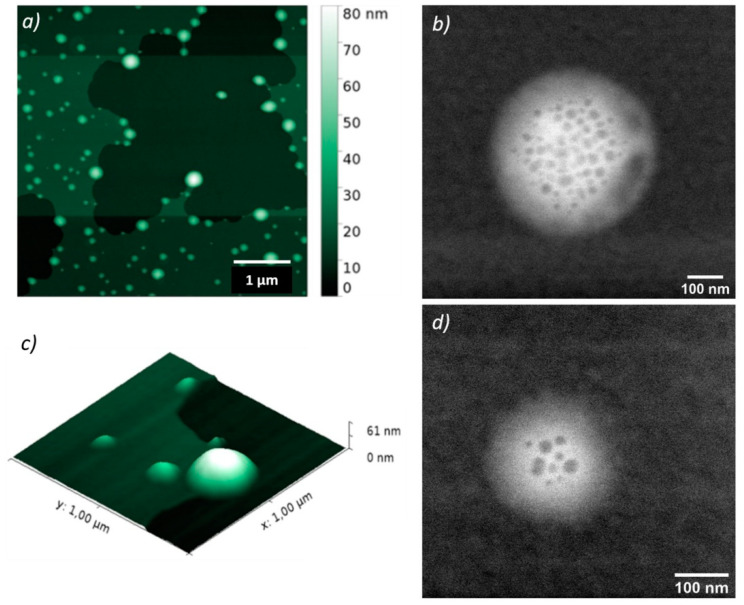
(**a**) AFM scans (5 × 5 µm2) of DOX_MagLipo dried on a silicon substrate. (**b**) Three-dimensional (3D) rendering of an enlargement of scan (**a**). (**c**,**d**) S/TEM images of DOX_MagLipo.

MagLipo and DOX-loaded MagLipo were prepared after hydration of the thin lipid film with a magnetic nanoparticles dispersion and extrusion through a porous membrane (Figure 1). AFM and S/TEM scans show the homogeneous spherical shape of nanocarriers (Figure 3).

Dynamic light scattering measurements revealed MagLipo and DOX_MagLipo nanometric dimensions with an average hydrodynamic diameter of 215.4 ± 42 nm and a surface zeta potential of −21.7 ± 5.1 mV with a polydispersity index (PDI) of around 0.2 (suggesting samples homogeneous dimension). Additionally, AFM scans confirmed nanometric dimensions, which resulted in being slightly underestimated with an average diameter of 177.6 ± 60.8 nm, because of particles dehydration after solvent evaporation that is caused by the drop casting of a liposomes water solution onto the silicon substrate prior to microscope scansion.

A negative value of zeta potential resulted from the incorporation of the DHSG lipid inside the bilayer of liposomes. Furthermore, a value of zeta potential below −20 mV guarantees good particles stability thanks to electrostatic repulsion. As this regard no irreversible aggregation of liposomes was observed, even after some weeks of storage at 4 °C, since, by just vortexing the dispersion, it has been possible to resuspend each trace of particles precipitation at the bottom of the vial. Moreover, the incorporation of DSPE-PEG_5000_ into nanoparticles bilayer contributes to liposome stability, avoiding particles aggregation, thanks to steric repulsion guaranteed by the long PEG chains [55]. After purification, the total lipid concentration was measured using the molar ratio used in the fabrication process resulting in an average lipid yield of 76 ± 0.6% and a cholesterol concentration of 0.28 ± 0.02 mg/mL. This evidence showed that a high fraction of the starting lipid quantity formed liposomes after the hydration and extrusion of the thin lipid film, confirming a good yield of the fabrication procedure. After purification with gel chromatography, DOX loading inside MagLipo was confirmed by fluorescence spectroscopy after the dissolution of lipid bilayer and an average drug concentration of 51.2 ± 24 µg/mL (94.2 ± 44.1 µM) was revealed.

**Figure 4:**
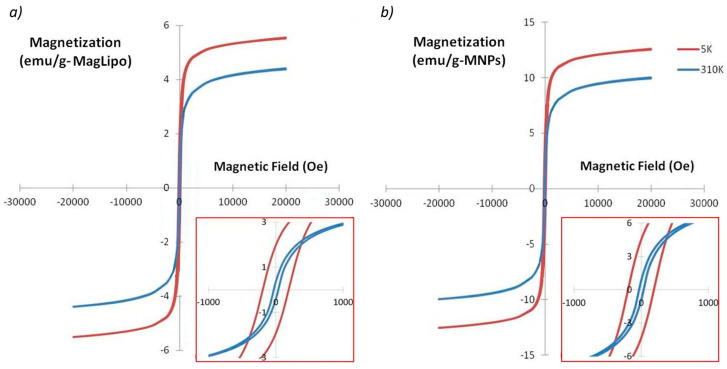
Magnetic characterization of MagLipo (**a**) and of MagNPs (**b**). Hysteresis curves acquired at 5 K and 310 K. In the inset enlargement of the plot showing hysteresis process.

Furthermore, successful MNPs encapsulation inside either MagLipo and DOX_MagLipo was demonstrated by S/TEM images, which clearly revealed the presence of iron oxide nanoparticles that were encapsulated inside liposomes (Figure 3b,d). MNPs concentration inside magnetic liposomes resulted of 0.48 ± 0.03 mg/mL after UV-Vis light spectroscopy analysis and, this value, as well as lipid concentration, was used in order to evaluate magnetization of liposomes (Figure 4).

The result of the magnetic characterization showed that the magnetization of the two materials are similar (Figure 4a,b), resulting with comparable magnetic properties. Magnetic properties at 310 K present an almost absent hysteresis, confirming the superparamagnetic behavior of encapsulated magnetic nanoparticles (Figure 4 inset). MagLipo present a higher coercive field, because of the encapsulation of magnetic nanoparticles inside the liposome. The magnetic saturation value of MagLipo is 4.2 emu/g at 310 K (9.8 emu/g for SPIONs), while 5.7 emu/g at 5 K (12.8 emu/g for SPIONs). Interestingly, the magnetization of magnetic liposomes is almost saturated with a low magnetic field, i.e., 10,000 Oe. This occurrence ensures an easy manipulation of magnetic liposomes by an external magnetic field.

### 3.2. Magnetic Targeting Experiments

#### 3.2.1. Selective Internalization of MagLipo in Cancer Cells: HepG2 Experiments

**Figure 5:**
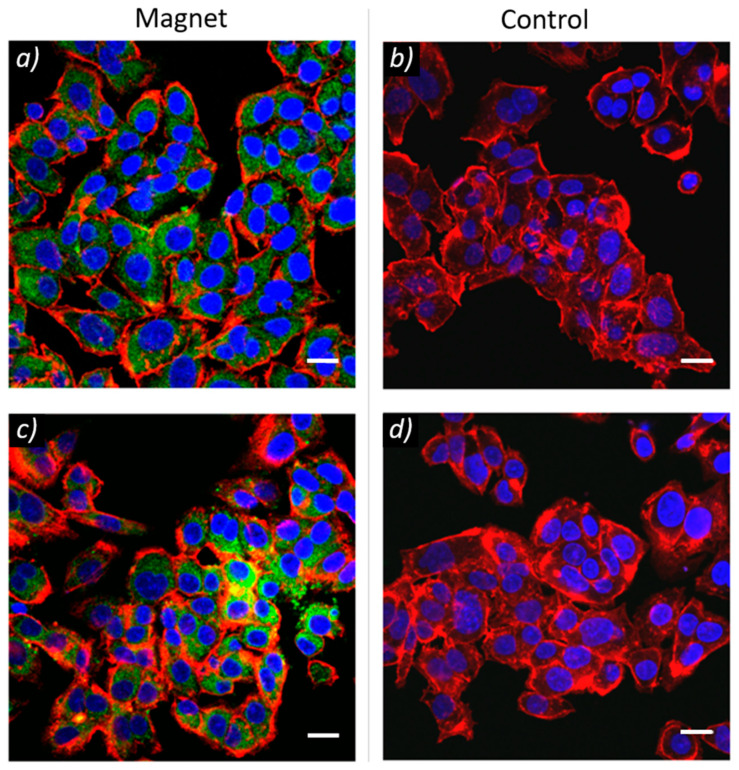
MagLipo magnetic targeting experiments showed selective liposomes cell internalization (in green) with HepG2 cancer cells in correspondence of the edge of the magnet (**a**,**c**). Conversely, no liposomes were present in the control channel, where no external magnet was placed (**b**,**d**). f-actin was stained in red, nuclei in blue. All scale bars are 20 µm.

Magnetic targeting experiments using HepG2 cancer cells were performed in order to assess the possibility to selectively enhance liposome internalization in cancer cells under flow conditions as described in Section 2. After 3 h with a constant flow of MagLipo-doped cell medium of about 2 mL/min., a strong uptake of MagLipo in HepG2 cells nearby the area of the microchannel exposed to the external magnetic field was revealed by confocal microscopy. Conversely, no MagLipo internalization in the control channel was revealed, which confirmed the high targeting efficiency for MagLipo (Figure 5) in a dynamic environment. Liposomes quantity inside HepG2 cell was calculated of 50.8 ± 3.1% of the average area occupied by cells in correspondence of the region of application of the external magnetic field, while that of the control channel was 0.40 ± 0.03%. No occlusion of both silicon tubing and microfluidic channel due to particle aggregation was observed during the experiments, confirming good colloidal stability of MagLipo. These experiments showed that the enhanced internalization of liposomes in cancer cells is possible, thanks to the presence of an adequate magnetic nanoparticles concentration that ensured MagLipo capture through an external magnetic field. MagLipo enhanced magnetic cell uptake reported in this study could have some benefits in reducing nanocarriers dispersion and clearance upon intravenous injection. Liposomes undergo a fast plasma clearance after 1–3 h from intravenous injection and maximum tumor uptake occurring in several hours [55,56]. Because the consistent MagLipo cancer cell uptake demonstrated in this experiment occurs within 3 h, our results support the possibility to reduce MagLipo plasma clearance upon injection and to speed up tumor nanoparticles internalization thanks to an external magnetic field.

#### 3.2.2. Analytical Description of Magnetic Targeting: HUVECs Experiments

**Figure 6:**
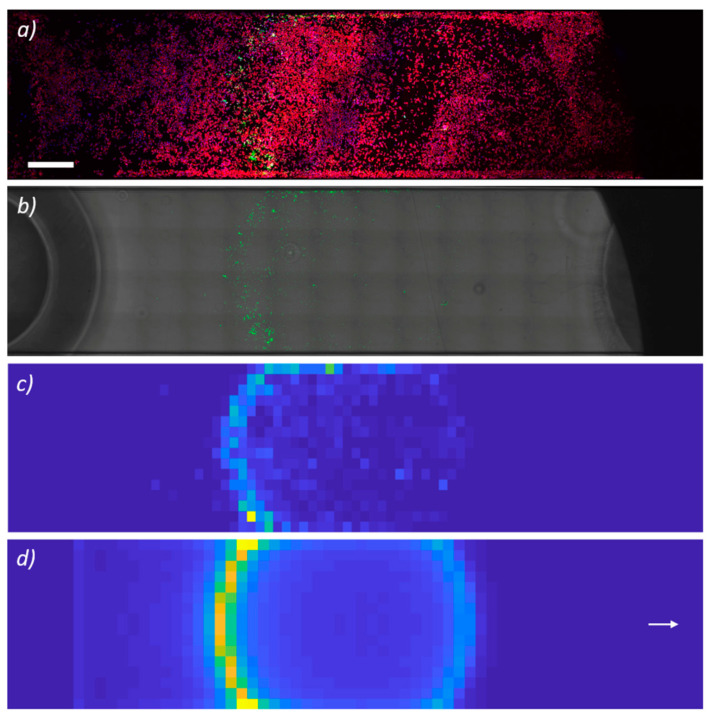
Confocal microscopy of the bottom side of the microchannel after human umbilical vein endothelial cells (HUVECs) staining (f-actin in red, nuclei in blue) (**a**). Fluorescent-labeled MagLipo (in green) are visible in a round-shaped area confirming selective magnetic cell internalization in correspondence of the edge of the external magnet. Distribution of fluorescence-labeled Mag-Lipo (in green), as attracted by the external magnetic field and internalized by HUVECs seeded at the bottom of the microchannel (bright field merged to MagLipo fluorescent signal) (**b**). (**c**) Density contour plot of the captured MagLipo, as obtained by fluorescence-based experimental recordings (**c**). Density contour plot of the captured MagLipo, as predicted by the numerical model; the arrow indicates flow direction (**d**). Flow velocity was set at 3.11 mL/min. Scale bar is 1 mm.

The distribution of MagLipo, as attracted by the external magnetic, is reported in Figure 6. In particular, Figure 6a displays microchannel reconstruction showing MagLipo (in green) selective internalization inside fluorescence-stained HUVECs, whereas Figure 6b shows the round-shaped contour of internalized MagLipo (in green) in correspondence of the edge of the external paled magnet. Moreover, Figure 6c,d feature the experimentally recorded and numerically predicted density contour plot of the captured MagLipo, respectively. The model remarkably predicted the experimental results, showing how internalization mostly occurred in correspondence of the magnet boundary (besides some layers close to the channel side boundary, where capture was favored by slow speed, i.e., viscous effects). This is in consistent with the fact that magnetic attraction is more pronounced close to the edge of the permanent magnet due to well-known field and gradient effects [57,58,59]. Overall, the possibility to accurately model the considered dynamic targeting strategy fosters the quantitative characterization and design of related approaches, in turn supporting potential translation to more clinically-relevant frameworks in the longer term.

All of the results concerning Magnetic Targeting demonstrated selective MagLipo enhanced cell internalization under various environmental conditions while using two different cell lines and highlighted the possibility of actively targeting the MagLipo cancer cell up-take and, consequently, of selectively localizing the effect of the encapsulated drug. Moreover, our evidences of efficient liposomes magnetic targeting could have an impact on the initial injected dose of DOX_MagLipo. As this regard, in vivo studies in humans on PEGylated DOX-loaded liposomes report on nanocarriers injected concentrations ranging from 10 to 80 mg/m^2^, depending on tumor type [56,57]. Because consistent MagLipo uptake has been shown in our experiments with a 10-fold less concentrated injected dose, this evidence suggests the feasibility to reduce liposomes injected amount with respect to conventional non-magnetic formulations.

### 3.3. Nanocarriers Internalization and Cytotoxicity Experiments on Hepatocellular Carcinoma Cell Line

**Figure 7:**
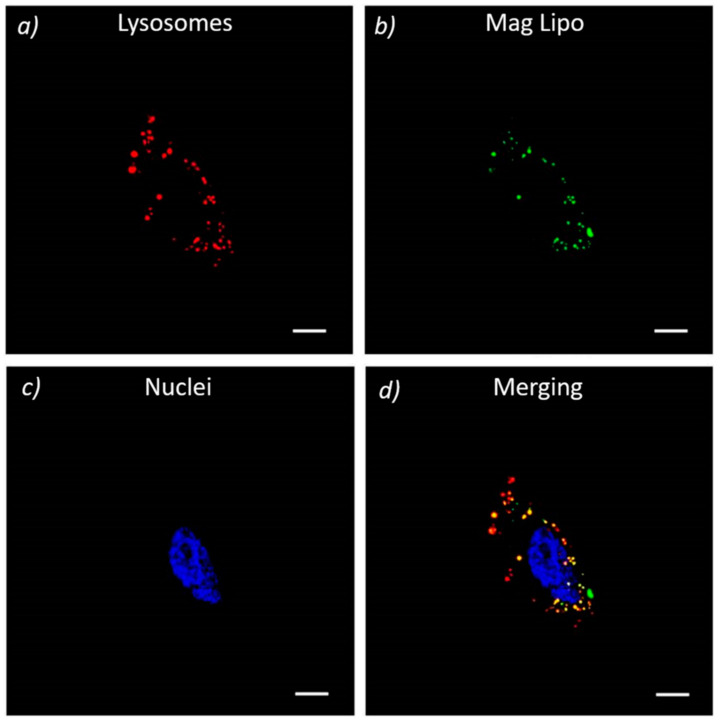
In vitro cell internalization of MagLipo in static conditions. Confocal fluorescence images of HepG2 cells showing MagLipo (in green) up-take and co-localization (in yellow) with lysosomes (in red) after LysoTracker assay; nuclei are stained in blue (All scale bars = 10 µm).

Confocal microscopy showed the strong uptake of MagLipo in HepG2 and red staining of lysosomes allowed for understanding particles localization inside the cells. In Figure 7, MagLipo are stained in green and overlapping is easily evidenced by the yellow signal that arises from the merging of the two channels, thus demonstrating successful internalization and lysosomes uptake of MagLipo in HepG2 cancer cells.

**Figure 8:**
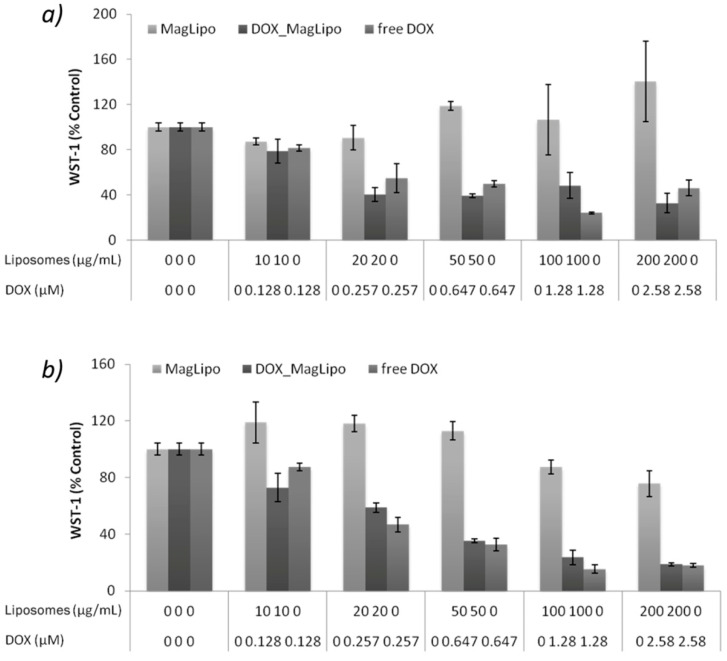
Metabolic WST-1 assay on HepG2 cancer cells after 24 h (**a**) and 72 h (**b**) of incubation with increasing concentrations of MagLipo, DOX_MagLipo, and free DOX.

The antitumor activities of MagLipo and DOX_MagLipo were investigated against HepG2 cells with the WST-1 viability assay and compared to that of the free-administered DOX. DOX_MagLipo inhibit cell viability after both 24 h (Figure 8a) and 72 h (Figure 8b) of incubation, as shown in Figure 8. In details, DOX_MagLipo showed a cytotoxic effect even after 24 h of incubation. In fact, for DOX_MagLipo concentrations of 20, 50, 100, and 200 µg/mL, corresponding to 0.257, 0.647, 1.28, and 2.58 µM of DOX, a viability reduction of about 50% was observed (Figure 8a). Furthermore, a dose-dependent anti-proliferative effect was instead observed after 72 h incubation (Figure 8b): at all of the tested concentrations, we reported a significant reduction of viability, which reaches about 80% of the control for the 200 µg/mL DOX_MagLipo concentration (2.58 µM of DOX). The free drug confirmed its antitumor action at both 24 h and at 72 h. Interestingly, its efficacy at 24 h is slightly lower with respect to DOX_MagLipo efficacy at liposomes concentration of 20, 50, and 200 µg/mL, underlying that liposomal encapsulation facilitates drug cell penetration upon liposomes cell uptake. As this regard, MagLipo internalization studies in HepG2 cancer cells showed high nanocarriers internalization and co-localization in lysosomes. This effect could be associated to the high cytotoxicity reported even after 24 h, since DOX release studies (see Supporting Information, Figure S1) showed consistent drug release at acidic pH proper of lysosomal environment [60]. Moreover, drug release studies showed slow DOX release at pH 7 over time, demonstrating the stability of liposomal formulation that prevents drug leaching prior to cell internalization.

Overall, these results indicated the safety and biocompatibility of the plain MagLipo. Conversely, DOX_MagLipo showed an even higher anti-proliferative effect against tumor cells that was induced by free DOX after 24 h, thus demonstrating enhancement of drug anticancer activity thanks to liposomal encapsulation.

## 4. Conclusions

The purpose of this work was to tackle the main issue that was encountered in systemic administration of chemotherapies against HCC: without an effective cancer targeting strategy, drugs undergo aspecific and uncontrolled biodistribution that leads to poor anticancer activity with consistent healthy tissue exposure, thus leading to severe toxic phenomena. As this regard, we presented a stable, superparamagnetic, and cytotoxic DOX_MagLipo formulation. Nanocarrier characterization showed nanometric dimensions, efficient MNPs, and DOX encapsulation and good colloidal stability of our formulation as well as superparamagnetic behavior confirmed by SQUID measurements that highlighted the good potential for magnetic manipulation. Moreover, we demonstrated enhanced nanocarriers in vitro cell uptake under cell medium flow in correspondence of a target area by the use of an external magnetic field with two different cell lines. HepG2 experiments confirmed remarkable and selective nanocarriers uptake in the target area, while HUVECs experiments validated the experimental data on MagLipo targeting capability. These results are significant, because they allowed correlating liposome targeting ability to constitutive parameters of nanocarriers (size, concentration, magnetization) and experimental conditions (channel dimensions, flow rate, viscosity, and temperature). This characterization ensured a predictable analysis of liposomes magnetic targeting, as well as a more comprehensive assessment of nanocarriers magnetic drug targeting. Remarkably, since the chosen dynamic conditions of Magnetic Targeting experiments are in good agreement with those that were reported in the literature for small blood vessels, our approach confirms the feasibility of pre-clinical evaluation of magnetic targeting and the possibility to accurately assess formulation parameters for further in vivo tests.

Furthermore, the high lysosomes uptake of MagLipo was confirmed by confocal microscopy, resulting in a fast DOX release inside the cell, which caused the high cytotoxicity of DOX_MagLipo towards HepG2 cancer cells, even after 24 h of incubation.

All of these results confirm the high therapeutic potential of DOX_MagLipo that could be exploited as an alternative to traditional chemotherapy against HCC. Furthermore, the encapsulation of MNPs with superparamagnetic behavior inside liposomes allows MRI tracking of our formulation during its biodistribution, envisioning the usage of DOX_MagLipo as theranostic platform with therapeutic and diagnostic capabilities. However, the preclinical representativeness of tumor invasion assays can be strengthened by using tumor spheroids models instead of single cells. Indeed, carrier transport in tumor tissue is affected by complex physical phenomena (including, e.g., interstitial flows, tissue heterogeneity, and carrier absorption/desorption dynamics) that, while posing modeling challenges [61,62], are being increasingly addressed through experimental microfluidic systems [63]. Thus, further in vitro investigations on advanced tumor models are needed to better assess the targeting efficacy of DOX_MagLipo, whence the potential for effective translation of our liposomal formulation to in vivo, clinically representative application scenarios. Finally, future investigations regarding the in vivo targeting efficacy of DOX_MagLipo will be carried out in order to verify the possible clinical translation of our liposomal formulation.

## Supplementary Materials

The following are available online at https://www.mdpi.com/2079-4991/10/11/2104/s1, Figure S1: DOX release kinetic from DOX_MagLipo under different environmental conditions; Figure S2: Confocal microscopy analysis of enhanced magnetic MagLipo cell targeting under pulsatile flow.

## References

[1] Balogh J., Victor D., Asham E.H., Burroughs S.G., Boktour M., Saharia A., Li X., Ghobrial R.M., Monsour H.P. (2016). Hepatocellular carcinoma: A review. J. Hepatocell. Carcinoma.

[2] El-Serag H.B., Rudolph K.L. (2007). Hepatocellular Carcinoma: Epidemiology and Molecular Carcinogenesis. Gastroenterology.

[3] Ghouri Y.A., Mian I., Rowe J.H. (2017). Review of hepatocellular carcinoma: Epidemiology, etiology, and carcinogenesis. J. Carcinog..

[4] Le Grazie M., Biagini M.R., Tarocchi M., Polvani S., Galli A. (2017). Chemotherapy for hepatocellular carcinoma: The present and the future. World J. Hepatol..

[5] Liu L., Cao Y., Chen C., Zhang X., McNabola A., Wilkie D., Wilhelm S., Lynch M., Carter C. (2006). Sorafenib blocks the RAF/MEK/ERK pathway, inhibits tumor angiogenesis, and induces tumor cell apoptosis in hepatocellular carcinoma model PLC/PRF/5. Cancer Res..

[6] Zhu A.X., Sahani D.V., Duda D.G., Di Tomaso E., Ancukiewicz M., Catalano O.A., Sindhwani V., Blaszkowsky L.S., Yoon S.S., Lahdenranta J. (2009). Efficacy, safety, and potential biomarkers of sunitinib monotherapy in advanced hepatocellular carcinoma: A phase II study. J. Clin. Oncol..

[7] Shaloam D., Tchounwou P.B. (2014). Cisplatin in cancer therapy: Molecular mechanisms of action. Eur. J. Pharmacol..

[8] Tan C.K., Chow P.K., Findlay M., Wong C., Machin D. (2000). Use of tamoxifen in hepatocellular carcinoma: A review and paradigm shift. J. Gastroenterol. Hepatol..

[9] Prajapati H.J., Dhanasekaran R., El-Rayes B.F., Kauh J.S., Maithel S.K., Chen Z., Kim H.S. (2013). Safety and efficacy of doxorubicin drug-eluting bead transarterial chemoembolization in patients with advanced hepatocellular carcinoma. J. Vasc. Interv. Radiol..

[10] Xu L., Xu S., Wang H., Zhang J., Chen Z., Pan L., Wang J., Wei X., Xie H., Zhou L. (2018). Enhancing the Efficacy and Safety of Doxorubicin against Hepatocellular Carcinoma through a Modular Assembly Approach: The Combination of Polymeric Prodrug Design, Nanoparticle Encapsulation, and Cancer Cell-Specific Drug Targeting. ACS Appl. Mater. Interfaces.

[11] Lohitesh K., Chowdhury R., Mukherjee S. (2018). Resistance a major hindrance to chemotherapy in hepatocellular carcinoma: An insight. Cancer Cell Int..

[12] Shi J., Kantoff P.W., Wooster R., Farokhzad O.C. (2014). Cancer nanomedicine: Progress, challenges and opportunities. Nat. Rev. Cancer.

[13] Hossen S., Hossain M.K., Basher M.K., Mia M.N.H., Rahman M.T., Uddin M.J. (2019). Smart nanocarrier-based drug delivery systems for cancer therapy and toxicity studies: A review. J. Adv. Res..

[14] Sun Q., Zhou Z., Qiu N., Shen Y. (2017). Rational Design of Cancer Nanomedicine: Nanoproperty Integration and Synchronization. Adv. Mater..

[15] Peer D., Karp J.M., Hong S., Farokhzad O.C., Margalit R., Langer R. (2007). Nanocarriers as an emerging platform for cancer therapy. Nat. Nanotechnol..

[16] Riehemann K., Schneider S.W., Luger T.A., Godin B., Ferrari M., Fuchs H. (2009). Nanomedicine-Challenge and Perspectives. Angew. Chemie Int. Ed..

[17] Genchi G.G., Marino A., Grillone A., Pezzini I., Ciofani G. (2017). Remote Control of Cellular Functions: The Role of Smart Nanomaterials in the Medicine of the Future. Adv. Healthc. Mater..

[18] Marino A., Camponovo A., Degl’Innocenti A., Bartolucci M., Tapeinos C., Martinelli C., De Pasquale D., Santoro F., Mollo V., Arai S. (2019). Multifunctional temozolomide-loaded lipid superparamagnetic nanovectors: Dual targeting and disintegration of glioblastoma spheroids by synergic chemotherapy and hyperthermia treatment. Nanoscale.

[19] Tapeinos C., Marino A., Battaglini M., Migliorin S., Brescia R., Scarpellini A., De Julián Fernández C., Prato M., Drago F., Ciofani G. (2019). Stimuli-responsive lipid-based magnetic nanovectors increase apoptosis in glioblastoma cells through synergic intracellular hyperthermia and chemotherapy. Nanoscale.

[20] Allen T.M., Cullis P.R. (2013). Liposomal drug delivery systems: From concept to clinical applications. Adv. Drug Deliv. Rev..

[21] Bulbake U., Doppalapudi S., Kommineni N., Khan W. (2017). Liposomal formulations in clinical use: An updated review. Pharmaceutics.

[22] Alexis F., Rhee J.-W., Richie J.P., Radovic-Moreno A.F., Langer R., Farokhzad O.C. (2008). New frontiers in nanotechnology for cancer treatment. Urol. Oncol..

[23] Owens D.E., Peppas N.A. (2006). Opsonization, biodistribution, and pharmacokinetics of polymeric nanoparticles. Int. J. Pharm..

[24] Wilhelm S., Tavares A.J., Dai Q., Ohta S., Audet J., Dvorak H.F., Chan W.C. (2016). Analysis of nanoparticles delivery to tumors. Nat. Rev. Mater..

[25] Kulkarni S.A., Feng S.S. (2013). Effects of particle size and surface modification on cellular uptake and biodistribution of polymeric nanoparticles for drug delivery. Pharm. Res..

[26] Aggarwal P., Hall J.B., McLeland C.B., Dobrovolskaia M.A., McNeil S.E. (2009). Nanoparticle interaction with plasma proteins as it relates to particle biodistribution, biocompatibility and therapeutic efficacy. Adv. Drug Deliv. Rev..

[27] Tapeinos C., Tomatis F., Battaglini M., Larrañaga A., Marino A., Telleria I.A., Angelakeris M., Debellis D., Drago F., Brero F. (2019). Cell Membrane-Coated Magnetic Nanocubes with a Homotypic Targeting Ability Increase Intracellular Temperature due to ROS Scavenging and Act as a Versatile Theranostic System for Glioblastoma Multiforme. Adv. Healthc. Mater..

[28] Lu Y.J., Chuang E.Y., Cheng Y.H., Anilkumar T.S., Chen H.A., Chen J.P. (2019). Thermosensitive magnetic liposomes for alternating magnetic field-inducible drug delivery in dual targeted brain tumor chemotherapy. Chem. Eng. J..

[29] Pradhan P., Giri J., Rieken F., Koch C., Mykhaylyk O., Döblinger M., Banerjee R., Bahadur D., Plank C. (2010). Targeted temperature sensitive magnetic liposomes for thermo-chemotherapy. J. Control. Release.

[30] Bothun G.D., Lelis A., Chen Y., Scully K., Anderson L.E., Stoner M.A. (2011). Multicomponent folate-targeted magnetoliposomes: Design, characterization, and cellular uptake. Nanomedicine.

[31] Ding X., Cai K., Luo Z., Li J., Hu Y., Shen X. (2012). Biocompatible magnetic liposomes for temperature triggered drug delivery. Nanoscale.

[32] Mody V.V., Cox A., Shah S., Singh A., Bevins W., Parihar H. (2014). Magnetic nanoparticle drug delivery systems for targeting tumor. Appl. Nanosci..

[33] Lübbe A.S., Alexiou C., Bergemann C. (2001). Clinical Applications of Magnetic Drug Targeting. J. Surg. Res..

[34] Centelles M.N., Wright M., So P.W., Amrahli M., Xu X.Y., Stebbing J., Miller A.D., Gedroyc W., Thanou M. (2018). Image-guided thermosensitive liposomes for focused ultrasound drug delivery: Using NIRF-labelled lipids and topotecan to visualise the effects of hyperthermia in tumours. J. Control. Release.

[35] Shah S.A., Aslam Khan M.U., Arshad M., Awan S.U., Hashmi M.U., Ahmad N. (2016). Doxorubicin-loaded photosensitive magnetic liposomes for multi-modal cancer therapy. Coll. Surf. B Biointerfaces.

[36] Dwivedi P., Kiran S., Han S., Dwivedi M., Khatik R., Fan R., Mangrio F.A., Du K., Zhu Z., Yang C. (2020). Magnetic Targeting and Ultrasound Activation of Liposome-Microbubble Conjugate for Enhanced Delivery of Anticancer Therapies. ACS Appl. Mater. Interfaces.

[37] Wang X., Yan F., Liu X., Wang P., Shao S., Sun Y., Sheng Z., Liu Q., Lovell J.F., Zheng H. (2018). Enhanced drug delivery using sonoactivatable liposomes with membrane-embedded porphyrins. J. Control. Release.

[38] Redolfi Riva E., Pastoriza-Santos I., Lak A., Pellegrino T., Pérez-Juste J., Mattoli V. (2017). Plasmonic/magnetic nanocomposites: Gold nanorods-functionalized silica coated magnetic nanoparticles. J. Colloid Interface Sci..

[39] Shen S., Huang D., Cao J., Chen Y., Zhang X., Guo S., Ma W., Qi X., Ge Y., Wu L. (2019). Magnetic liposomes for light-sensitive drug delivery and combined photothermal-chemotherapy of tumors. J. Mater. Chem. B.

[40] Zangabad P.S., Mirkiani S., Shahsavari S., Masoudi B., Masroor M., Hamed H., Jafari Z., Taghipour Y.D., Hashemi H., Karimi M. (2018). Stimulus-responsive liposomes as smart nanoplatforms for drug delivery applications. Nanotechnol. Rev..

[41] Ferjaoui Z., Jamal Al Dine E., Kulmukhamedova A., Bezdetnaya L., Soon Chang C., Schneider R., Mutelet F., Mertz D., Begin-Colin S., Quilès F. (2019). Doxorubicin-Loaded Thermoresponsive Superparamagnetic Nanocarriers for Controlled Drug Delivery and Magnetic Hyperthermia Applications. ACS Appl. Mater. Interfaces.

[42] Schleich N., Danhier F., Préat V. (2015). Iron oxide-loaded nanotheranostics: Major obstacles to in vivo studies and clinical translation. J. Control. Release.

[43] Martina M., Wilhelm C., Lesieur S. (2008). The effect of magnetic targeting on the uptake of magnetic-fluid-loaded liposomes by human prostatic adenocarcinoma cells. Biomaterials.

[44] Rȩkorajska A., Szuplewska A., Rȩkorajska Joniec A., Pocztańska E., Krysiński P., Dybko A., Chudy M. (2019). Magnetic field-assisted selective delivery of doxorubicin to cancer cells using magnetoliposomes as drug nanocarriers. Nanotechnology.

[45] Lammers T., Kiessling F., Hennink W.E., Storm G. (2012). Drug targeting to tumors: Principles, pitfalls and (pre-) clinical progress. J. Control. Release.

[46] Youn Y.S., Bae Y.H. (2018). Perspectives on the past, present, and future of cancer nanomedicine. Adv. Drug Deliv. Rev..

[47] Hare J.I., Lammers T., Ashford M.B., Puri S., Storm G., Barry S.T. (2017). Challenges and strategies in anti-cancer nanomedicine development: An industry perspective. Adv. Drug Deliv. Rev..

[48] Del Turco S., Ciofani G., Cappello V., Parlanti P., Gemmi M., Caselli C., Ragusa R., Papa A., Battaglia D., Sabatino L. (2019). Effects of cerium oxide nanoparticles on hemostasis: Coagulation, platelets, and vascular endothelial cells. J. Biomed. Mater. Res. Part A.

[49] Cullis P.R., Hope M.J., Bally M.B., Madden T.D., Mayer L.D., Fenske D.B. (1997). Influence of pH gradients on the transbilayer transport of drugs, lipids, peptides and metal ions into large unilamellar vesicles. Biochim. Biophys. Acta Rev. Biomembr..

[50] Grillone A., Riva E.R., Mondini A., Forte C., Calucci L., Innocenti C., de Julian Fernandez C., Cappello V., Gemmi M., Moscato S. (2015). Active Targeting of Sorafenib: Preparation, Characterization, and In Vitro Testing of Drug-Loaded Magnetic Solid Lipid Nanoparticles. Adv. Healthc. Mater..

[51] Riva C.E., Grunwald J.E., Sinclair S.H., Petrig B.L. (1985). Blood velocity and volumetric flow rate in human retinal vessels. Investig. Ophthalmol. Vis. Sci..

[52] Williams C., Wick T.M. (2005). Endothelial cell-smooth muscle cell co-culture in a perfusion bioreactor system. Ann. Biomed. Eng..

[53] Grillone A., Battaglini M., Moscato S., Mattii L., De Julián Fernández C., Scarpellini A., Giorgi M., Sinibaldi E., Ciofani G. (2019). Nutlin-loaded magnetic solid lipid nanoparticles for targeted glioblastoma treatment. Nanomedicine.

[54] Berselli L.C., Miloro P., Menciassi A., Sinibaldi E. (2013). Exact solution to the inverse Womersley problem for pulsatile flows in cylindrical vessels, with application to magnetic particle targeting. Appl. Math. Comput..

[55] Dos Santos N., Allen C., Doppen A.M., Anantha M., Cox K.A.K., Gallagher R.C., Karlsson G., Edwards K., Kenner G., Samuels L. (2007). Influence of poly(ethylene glycol) grafting density and polymer length on liposomes: Relating plasma circulation lifetimes to protein binding. Biochim. Biophys. Acta Biomembr..

[56] Martin F., Huang A., Uziely B., Kaufman B., Safra T. (1994). Prolonged Circulation Time and Enhanced Accumulation in Malignant Exudates of Doxorubicin Encapsulated in Polyethylene-glycol Coated Liposomes. Cancer Res..

[57] Gabizon A., Shmeeda H., Barenholz Y. (2003). Pharmacokinetics of Pegylated Liposomal Doxorubicin: Review of Animal and Human Studies. Clin. Pharmacokinet..

[58] Subramanian M., Miaskowski A., Jenkins S.I., Lim J., Dobson J. (2019). Remote manipulation of magnetic nanoparticles using magnetic field gradient to promote cancer cell death. Appl. Phys. A Mater. Sci. Process..

[59] Cao Q., Han X., Li L. (2012). Numerical analysis of magnetic nanoparticle transport in microfluidic systems under the influence of permanent magnets. J. Phys. D. Appl. Phys..

[60] He X., Li J., An S., Jiang C. (2013). pH-sensitive drug-delivery systems for tumor targeting. Ther. Deliv..

[61] Pizzichelli G., Di Michele F., Sinibaldi E. (2016). An analytical model for nanoparticles concentration resulting from infusion into poroelastic brain tissue. Math. Biosci..

[62] Di Michele F., Pizzichelli G., Mazzolai B., Sinibaldi E. (2015). On the preliminary design of hyperthermia treatments based on infusion and heating of magnetic nanofluids. Math. Biosci..

[63] Huang Y.L., Ma Y., Wu C., Shiau C., Segall J.E., Wu M. (2020). Tumor spheroids under perfusion within a 3D microfluidic platform reveal critical roles of cell-cell adhesion in tumor invasion. Sci. Rep..

