# Supplementary material for: Enhanced In Vitro Magnetic Cell Targeting of Doxorubicin-Loaded Magnetic Liposomes for Localized Cancer Therapy
Source: Nanomaterials (Basel). 2020 Oct 23;10(11):2104. doi: 10.3390/nano10112104 (PMC7690690; doi:10.3390/nano10112104)
Supplement: Supplementary file 1 [file nanomaterials-10-02104-s001.pdf]

nanomaterials

Supplementary Materials

Drug release experiments

Drug release profile from DOX_MagLipo was evaluated by diffusion of the drug through a porous membrane. A plastic dialysis tube (membrane cutoff = 3.5 kDa) was stuck on the bottom cut of a 50 mL Falcon tube and filled with 500 µL of DOX-loaded magnetic liposome dispersion. The falcon tube was filled with 3 mL phosphate buffer saline (PBS) in order to have enough volume to wet the dialysis tube. This system was stored at 37 °C under gentle mechanical agitation. At various time intervals DOX signal inside the PBS was measured by fluorescence spectroscopy using a microplate reader (Victor3, Perkin Elmer), setting the excitation wavelength at 480 nm and by acquiring fluorescence signal from 560 to 590 nm. After each fluorescence measurement, PBS was discarded and the falcon tube was washed and refilled with 3 mL of fresh PBS. Cumulative DOX release was thus evaluated at three different pH (pH 3, 7, and 10) and drug concentration was correlated to fluorescence signal by means of a standard curve using the DOX solution in PBS with known concentration. The percentage of DOX released at each time interval was calculated as follows (equation 1):

$$\boldsymbol{DOX}_{\%tot} = \sum_{i=0}^{t-1} \boldsymbol{DOX}_{\%}(\boldsymbol{i}) + \boldsymbol{DOX}_{\%}(\boldsymbol{t}) \qquad (1)$$

where i is the sampling time of recorded DOX fluorescence and t is the actual time interval when DOX concentration is measured via fluorescence spectroscopy. NaOH and HCl was used to equilibrate pH of each DOX_MagLipo batch and PBS aliquots. All the experiments were repeated in triplicate.

Drug release behavior for DOX_MagLipo showed a faster DOX release for liposomes equilibrated in PBS at pH 3 with respect to those at pH 7 and 10. DOX release percentage revealed after 72 hours was about 13% for the batch equilibrated at pH 3, about 10% for the batch at pH 7 and about 2% for the batch at pH 10 (Figure S1). This effect is probably due to acidic conditions that cause a destabilization of liposome bilayer with consequent enhanced drug diffusion [1].

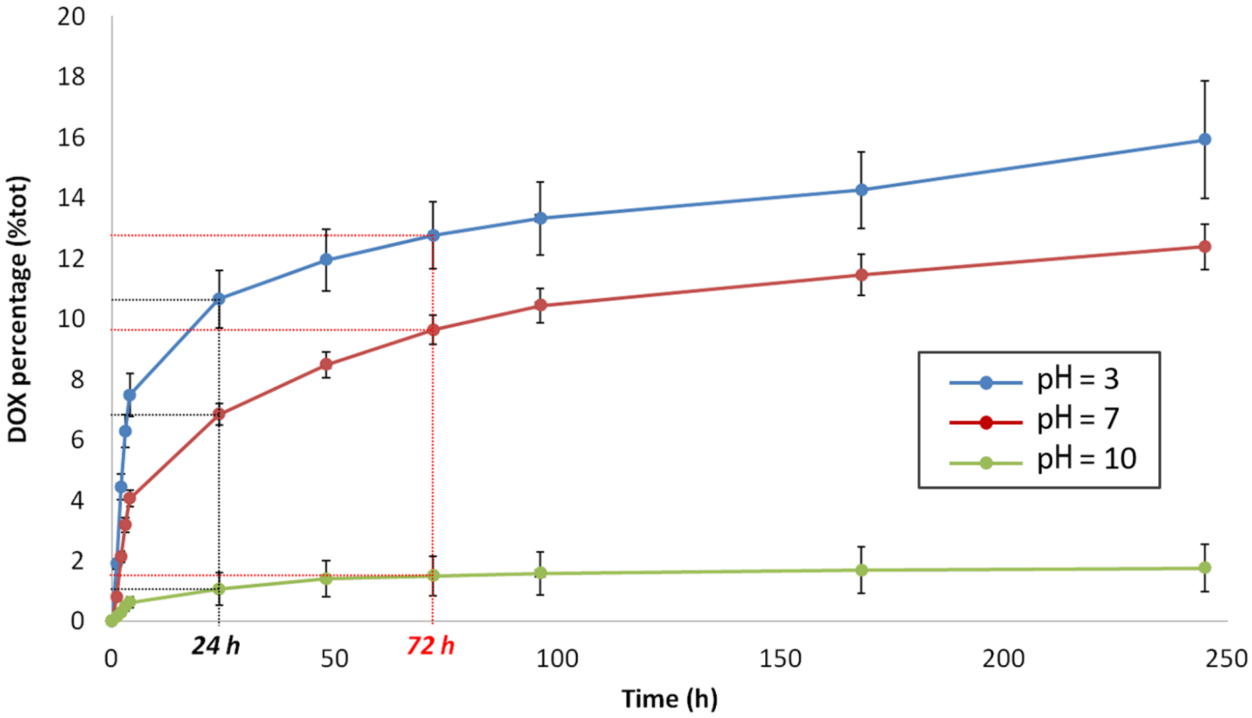

Figure 1. DOX release profile from DOX_MagLipo equilibrated in PBS at pH 3 (blue curve), pH 7 (red curve), pH 10 (green curve).

Moreover, drug solubility plays a role in the release kinetic, since higher solubility of DOX in water environment at acidic pH facilitate drug diffusion from liposomes bilayer [2] Conversely, DOX release in basic environments results in a slow kinetic, probably due to the difficulty in the diffusion of the drug from the bilayer membrane of liposome and to low water solubility of DOX in basic environments.

HUVECs magnetic targeting experiments under pulsatile and constant flow conditions

MagLipo targeting experiments under pulsatile flow conditions were performed for 5 hrs using a variable flow rate ranging between 0 and 5 mL/min with a periodicity of 1.2 s between each flow peak, corresponding to shear stress pulses of 0 and 96.1 dyn/cm2 respectively. HUVECs used for these experiments were seeded, washed and stained with the same detailed procedure explained in Materials and Methods section. For these experiments images of cell-internalized MagLipo were taken via confocal microscopy. Confocal microscopy showed that MagLipo enhanced HUVECs internalization was possible even under pulsatile cell medium flow conditions, since a MagLipo quantity of 3.7 ± 0.9 % of the average area occupied by cells was revealed. This low liposomes quantity calculated for pulsatile flow experiments is probably due to a more complex fluid dynamics, which will be further investigated in a different study. Anyhow, this experiment showed that enhanced cell targeting is possible even under more complex fluid dynamics, highlighting the possibility to using these nanocarriers in a future clinical scenario for localized anticancer therapy.

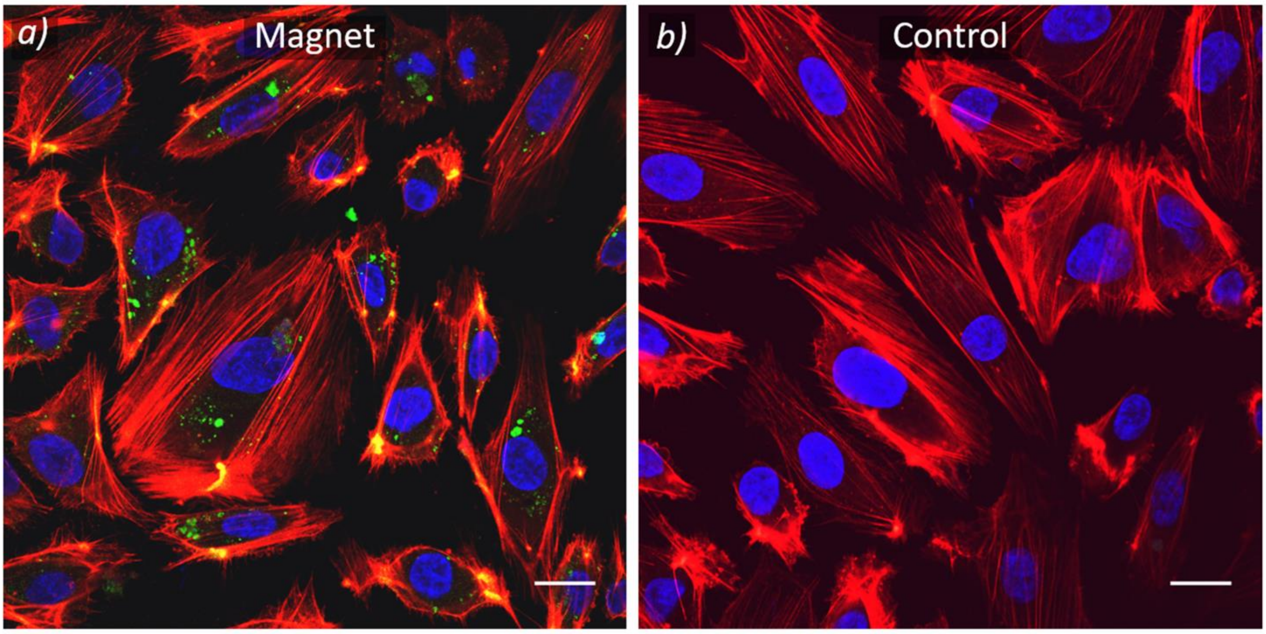

Figure 2. MagLipo magnetic targeting experiments under pulsatile flow conditions showed selective liposomes cell internalization in HUVECs in correspondence of the magnet position. Conversely, no liposomes were present in the control channel, where no external magnet have been placed. All scale bars are of 20 μm.

References

1. Manconi, M.; Isola, R.; Falchi, A.M.; Sinico, C.; Fadda, A.M. Intracellular distribution of fluorescent probes delivered by vesicles of different lipidic composition. *Colloids Surfaces B Biointerfaces* **2007**, *57*, 143–151, doi:10.1016/j.colsurfb.2007.01.016.

2. Gillies, E.R.; Fréchet, J.M.J. pH-responsive copolymer assemblies for controlled release of doxorubicin. *Bioconjug. Chem.* **2005**, *16*, 361–368, doi:10.1021/bc049851c.
